# Methotrexate and the platelet count.

**DOI:** 10.1038/bjc.1968.31

**Published:** 1968-06

**Authors:** D. Ogston, A. A. Dawson, J. F. Philip


### METHOTREXATE AND THE PLATELET COUNT

D. OGSTON, A. A. DAWSON AND J. F. PHILIP

*From the Department of Medicine, University of Aberdeen, and the Malignant Diseases Unit, Royal Infirmary, Aberdeen*

Received for publication December 27, 1967

DEPRESSION of the haemopoietic system with leucopenia and thrombocytopenia is a well-recognised hazard of the use of antimitotic agents in the treatment of malignant disease. During a study of the causes of thrombocytosis, however, we observed that a rise in the platelet count appeared to be frequent following methotrexate therapy. We report our findings on the effect of methotrexate on the platelet count, and, in particular, on its effect in producing a thrombocytosis.

### PATIENTS AND METHODS

The case records of 39 patients who had received methotrexate (amethopterin) as therapy for malignant disease were examined. Serial platelet counts were performed, at intervals of not more than 3 days, before, throughout, and after the period of therapy.

Methotrexate was administered by intra-arterial infusion after cannulation of the regional artery supplying the site of the tumour (18 patients) by intravenous injections (19 patients) or orally (2 patients). The total methotrexate dose by intra-arterial infusion ranged from 150 mg. to 640 mg. given over a period of 3 to 16 days, mean 7·0 days; by the intravenous route, 15 mg. to 150 mg. over 3–27 days, mean 8·5 days; and the oral dosages were 70 mg. and 110 mg., over 15 and 25 days, respectively. The length of therapy exceeded 10 days in only 9 patients.

The malignant conditions treated were carcinomas of breast (13); mouth, fauces or tongue (7); larynx (4); skin (4); oesophagus (3); vulva (3); jaw (2); parotid gland (1); bronchus (1); ear (1). Although many patients had recurrent carcinomas, especially of the squamous epithelial type, none had evidence of disseminated disease beyond the regional lymph nodes clinically, and none had a leucoerythroblastic blood picture before therapy. Bone-marrow biopsy was not carried out as a routine procedure.

Platelet counts were performed by the method of Oettle and Spriggs (1951). The normal range of the platelet count in our laboratory is 150–270,000 c.mm.

### RESULTS

*Phase of thrombocytopenia*—Table I shows that a reduction in the platelet count of more than 25% of the pre-treatment level occurred in 31 of the 39 patients. It is also apparent that the platelet count fell slightly earlier after both the start and the end of the course of intra-arterial, than after intravenous, administration of methotrexate, presumably because of the higher dosage in the intra-arterial treated group.

TABLE I.—*Fall in the Platelet Count with Methotrexate Therapy*

| | | Mode of administration | | | |
|---|---|---|---|---|---|
| | | Intra-arterial | Intra-venous | Oral | Total |
| | | Number of patients | | | |
| Maximum fall in platelet count, as % of pre-treatment count | (a) Less than 25% | 4 | 3 | 1 | 8 |
| | (b) 25–49% | 5 | 4 | 1 | 10 |
| | (c) 50–74% | 5 | 8 | — | 13 |
| | (d) More than 75% | 4 | 4 | — | 8 |
| Time of maximum fall in groups (b), (c) and (d) from start of therapy (days) | 7–10 | 8 | 1 | — | 9 |
| | 11–14 | 5 | 7 | — | 12 |
| | 15–18 | 1 | 8 | 1 | 10 |
| Time of maximum fall in groups (b), (c) and (d) from end of therapy (days) | Before end of therapy | 1 | 1 | — | 2 |
| | 0–3 | 7 | 2 | 1 | 10 |
| | 4–7 | 6 | 10 | — | 16 |
| | 8–11 | — | 3 | — | 3 |

*Phase of thrombocytosis.*—After the period when the majority of patients showed thrombocytopenia, a rise in the platelet count of over 25% above the pre-treatment level was observed in 37 of the 39 subjects studied (Fig. 1). In 29 of these, this rise followed a fall in the platelet count of at least 25% of the initial level. In 25 patients the maximum platelet count exceeded 500,000 c.mm. It is evident from Table II that the peak of this thrombocytosis occurs at a fairly constant time after the start of therapy, the end of therapy, and the minimum platelet count, with both intra-arterial and intravenous therapy.

The rise in the platelet count bore no relationship to recognised intercurrent infection, or to operative procedures during the period of study. Twenty-two patients had started radiotherapy before the maximum platelet count, but the time between the start of radiotherapy and the maximum platelet count varied

TABLE II.—*Rise in the Platelet Count After Methotrexate*

| | | Mode of administration | | | |
|---|---|---|---|---|---|
| | | Intra-arterial | Intra-venous | Oral | Total |
| | | Number of patients | | | |
| All patients with rise in platelet count | | 18 | 18 | 1 | 37 |
| Time between start of therapy and maximum platelet count (days) | Less than 15 | — | 1 | — | 1 |
| | 15–18 | 4 | 1 | — | 5 |
| | 19–22 | 12 | 9 | 1 | 22 |
| | 23–26 | 2 | 2 | — | 4 |
| | More than 27 | — | 5 | — | 5 |
| Time between end of therapy and maximum platelet count (days) | On therapy | — | — | 1 | 1 |
| | Less than 10 | 1 | — | — | 1 |
| | 10–13 | 7 | 6 | — | 13 |
| | 14–17 | 9 | 10 | — | 19 |
| | 18–21 | 1 | 2 | — | 3 |
| Patients with fall in platelet count followed by rise | | 14 | 15 | — | 29 |
| Time between minimum and maximum platelet counts (days) | 3–6 | — | 2 | — | 2 |
| | 7–10 | 10 | 10 | — | 20 |
| | 11–14 | 4 | 3 | — | 7 |

from 1 to 18 days, and radiotherapy did not appear to influence the extent or timing of the thrombocytosis.

Eighteen patients with a rise in the platelet count of over 50% had serial counts continued long enough to allow assessment of the period of thrombocytosis. Twelve of these patients started radiotherapy, either before the peak of the platelet count, or before its fall to around the initial level. This did not substantially

FIG. 1.—Pre-treatment, minimum and maximum platelet counts after start of methotrexate therapy.

reduce the duration of thrombocytosis, the mean period of an increased platelet count being 12·8 days in the 12 patients, compared with 13·5 days in the 6 patients not having radiotherapy. Table III shows the lack of correlation between the extent and duration of the rise in platelet count.

TABLE III.—*Length of Period of Increased Platelet Count*

| Maximum rise in platelet count (% of pre-treatment count) | Number of patients | Mean period of increased platelet count (days) |
|---|---|---|
| 50–99% | 4 | 12·3 |
| 100–199% | 9 | 12·9 |
| More than 200% | 5 | 14·0 |

*Changes in the leucocyte count*

Twenty-five of the 39 patients developed leucopenia (fall in the W.B.C. count to below 3,500 c.mm.) during or following methotrexate therapy. The time for the maximum fall in the leucocyte count showed greater variation than that for platelets, but the majority occurred in the same time period; 7 to 16 days after the start of therapy. Nineteen of the 25 leucopenic patients had a fall in platelet count to below 100,000 c.mm., and 23 had a reduction in the platelet count exceeding 25%.

The records were examined to determine whether a leucocytosis developed in conjunction with a rise in the platelet count. Twelve patients had a W.B.C. count exceeding 10,000 c.mm., but the time when this occurred ranged from one to 44 days after the start of therapy, and in the majority, the rise was clearly related to infection or an operative procedure. There was no relationship between the timing of the leucocytosis and that of the thrombocytosis.

*Changes in the platelet count following other anti-mitotic agents*

The records of 8 patients who had serial platelet counts following anti-mitotic therapy other than methotrexate were examined. Six had a reduction in the platelet count exceeding 25% and 5 had a later rise in the platelet count exceeding 25% of the pre-treatment level. The pre-treatment, minimum and maximum counts in the 8 patients are shown in Fig. 2. It can be seen that the majority had the same sequence as observed for methotrexate, but the changes were less consistent, and tended to occur earlier.

### DISCUSSION

We have shown that the administration of therapeutic doses of methotrexate is followed, in the majority of patients, by a reduction in the platelet count with a subsequent thrombocytosis; this latter change has not been reported previously in relation to anti-mitotic therapy, though the occurrence of thrombocytosis in malignant disease is well documented (Levin and Conley, 1964). Whether the thrombocytosis is an effect of methotrexate *per se* or a response to a period of thrombocytopenia is not clear. It seems unlikely that a marrow-depressing drug should cause delayed stimulation of one of the marrow elements; a " rebound " phenomenon following thrombocytopenia appears to be a more satisfactory explanation. However, some patients developed thrombocytosis in the absence of an observed preceding thrombocytopenia, and there was no relationship between

FIG. 2.—Pre-treatment, minimum and maximum platelet counts after start of anti-mitotic therapy the severity of the thrombocytopenia and the extent of the succeeding thrombocytosis, but it is possible that a very transient phase of thrombocytopenia was missed in some patients. While few patients treated with other anti-mitotic drugs had platelet counts performed for a sufficiently long period after cessation of therapy to determine whether the same sequence occurs as after methotrexate, the findings in the small number of patients studied suggest that the production of thrombocytopenia followed by a thrombocytosis is not confined to methotrexate.

It is recognised that replacement therapy for vitamin $B_{12}$ and folic acid deficiency states may be accompanied by a thrombocytosis and it is possible that cessation of treatment with a folic acid antagonist produces the same change. There is now, however, considerable evidence that, under certain circumstances, human plasma contains a substance (" thrombopoietin ") capable of promoting platelet production (Schulman *et al.*, 1960). Removal or destruction of platelets in the rat stimulates increased platelet production with a subsequent rise in the platelet count to above the initial level (Matter *et al.*, 1960) and it has been suggested that this is mediated through the release of " thrombopoietin " (de Gabriele and Pennington, 1967). It seems likely that the thrombocytosis following methotrexate therapy is due to the release of " thrombopoietin " during the period of thrombocytopenia rather than to a specific effect of methotrexate.

### SUMMARY

The thrombocytopenia resulting from methotrexate therapy is frequently followed by a thrombocytosis. It is postulated that the thrombocytosis is due to non-specific stimulation of platelet production resulting from thrombocytopenia rather than to a specific effect of methotrexate *per se*.

### REFERENCES

DE GABRIELE, G. AND PENNINGTON, D. G.—(1967) *Br. J. Haemat.*, **13**, 210.
LEVIN, J. AND CONLEY, C. L.—(1964) *Archs intern. Med.*, **114**, 497.
MATTER, M. HARTMANN, J. R. KAUTZ, J, DEMARSH, Q. B. AND FINCH, C. A.—(1960) *Blood* **15**, 174.
OETTLE, A. G. AND SPRIGGS, A. I.—(1951) ' Recent Advances in Clinical Pathology. ' 2nd Edition. London (J. & A. Churchill), p. 406.
SCHULMAN, I., PIERCE, M., LUKENS, A. AND CURRIMBHOY, Z.—(1960) *Blood*, **16**, 943.


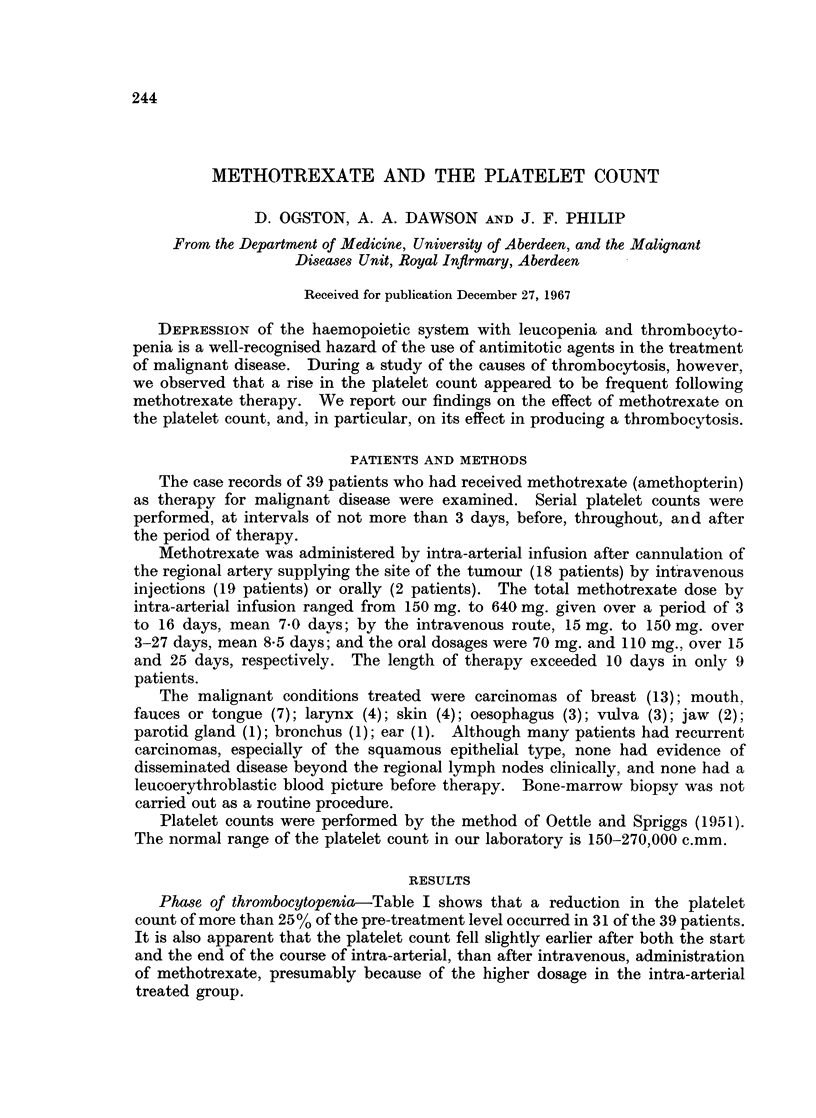

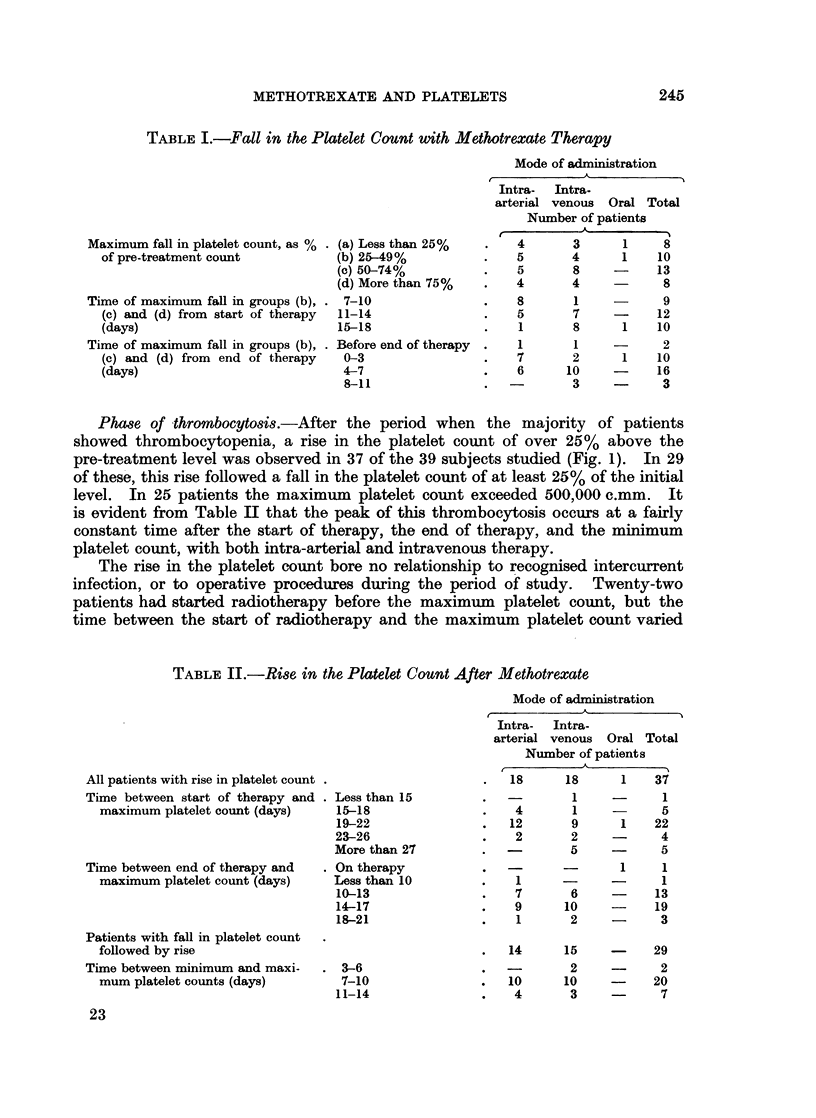

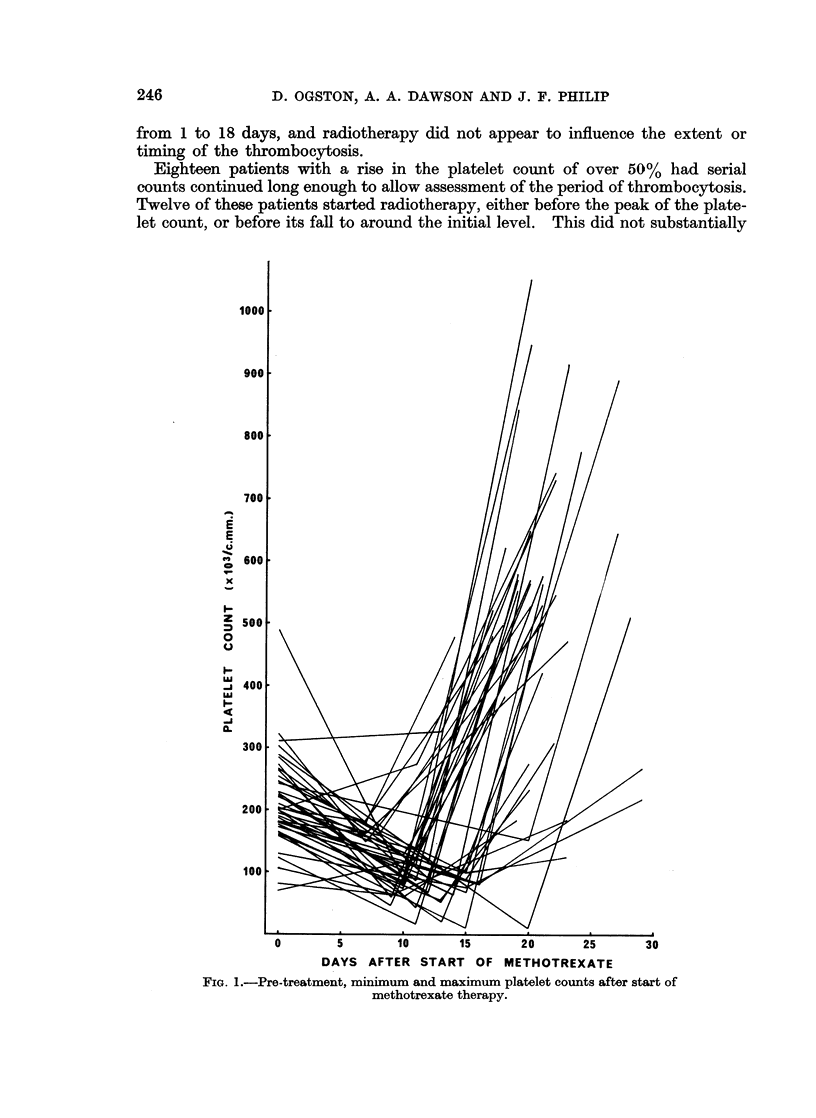

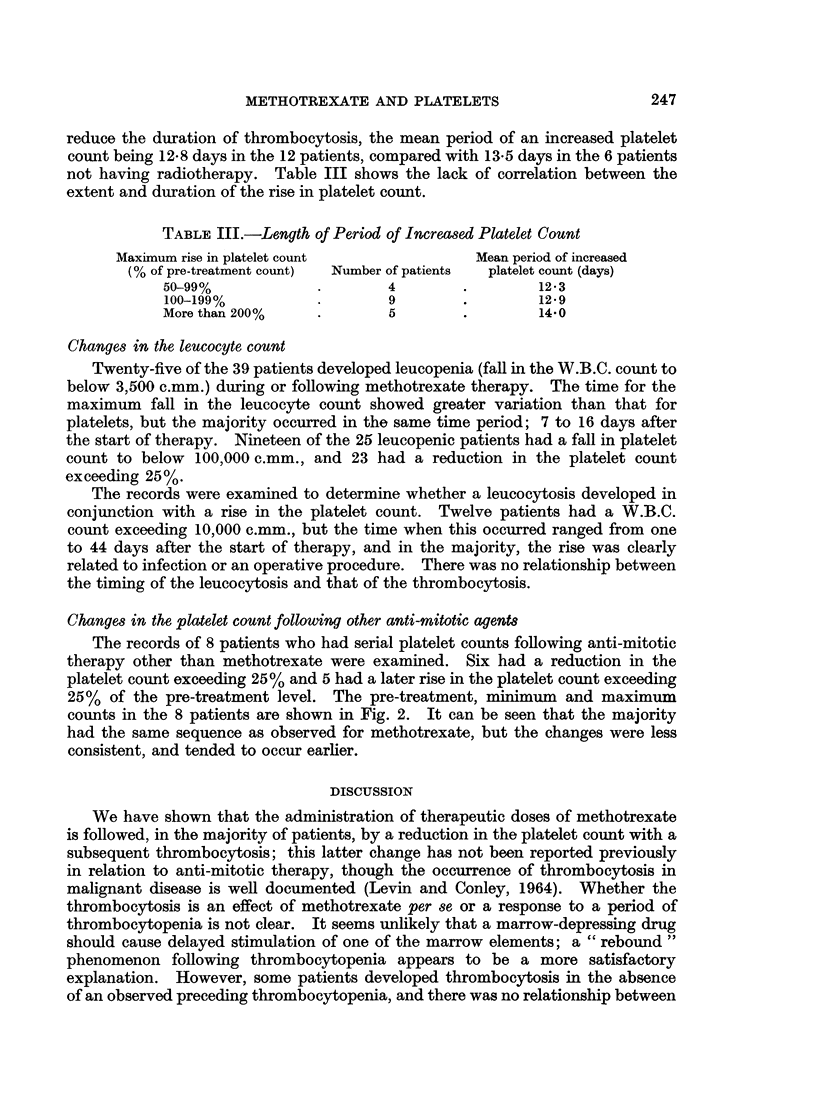

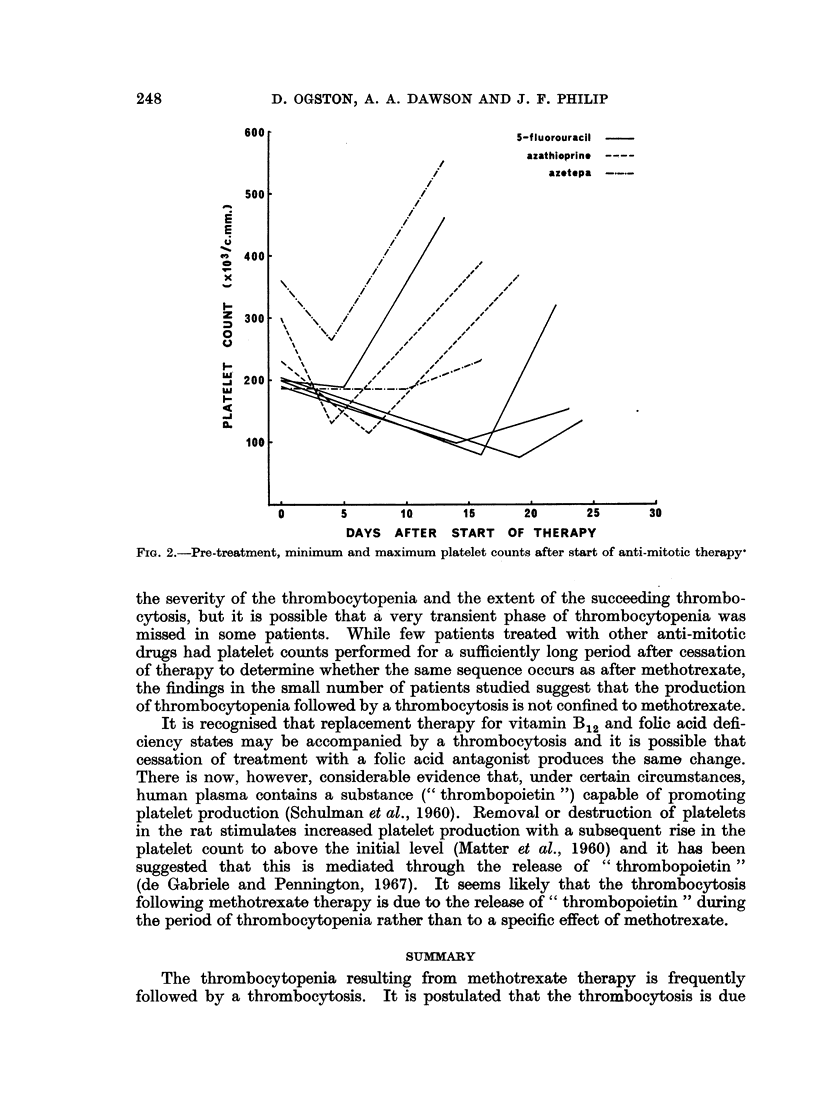

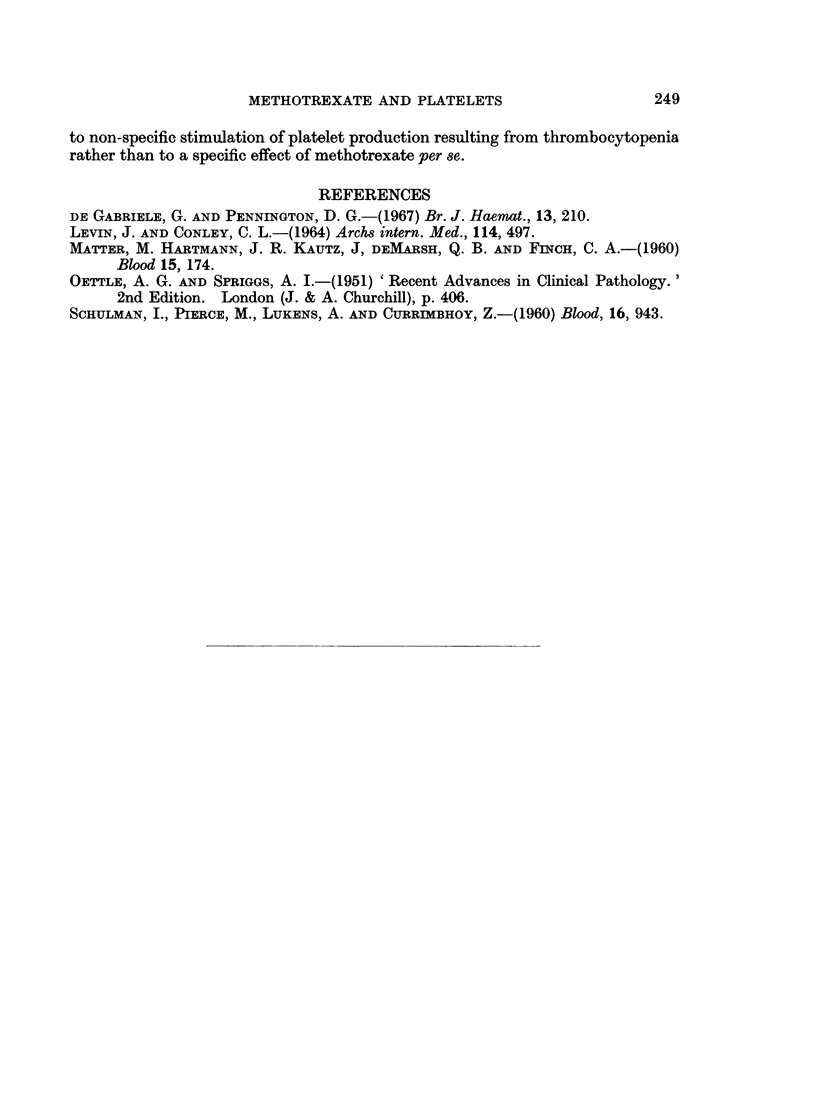


## References

[OCR_00462] LEVIN J., CONLEY C. L. (1964). THROMBOCYTOSIS ASSOCIATED WITH MALIGNANT DISEASE.. Arch Intern Med.

[OCR_00472] SCHULMAN I., PIERCE M., LUKENS A., CURRIMBHOY Z. (1960). Studies on thrombopoiesis. I. A factor in normal human plasma required for platelet production; chronic thrombocytopenia due to its deficiency.. Blood.

